# Highly sensitive and quantitative evaluation of the EGFR T790M mutation by nanofluidic digital PCR

**DOI:** 10.18632/oncotarget.4058

**Published:** 2015-05-10

**Authors:** Eiji Iwama, Koichi Takayama, Taishi Harada, Isamu Okamoto, Fumihiko Ookubo, Junji Kishimoto, Eishi Baba, Yoshinao Oda, Yoichi Nakanishi

**Affiliations:** ^1^ Faculty of Medical Sciences, Department of Comprehensive Clinical Oncology, Kyushu University, Fukuoka, Japan; ^2^ Research Institute for Diseases of the Chest, Graduate School of Medical Sciences, Kyushu University, Fukuoka, Japan; ^3^ Center for Clinical and Translational Research, Kyushu University Hospital, Fukuoka, Japan; ^4^ Division of Diagnostic Pathology, Kyushu University Hospital, Fukuoka, Japan; ^5^ Department of Research and Development of Next Generation Medicine, Kyushu University, Fukuoka, Japan; ^6^ Department of Anatomic Pathology, Graduate School of Medical Sciences, Kyushu University, Fukuoka, Japan

**Keywords:** EGFR, T790M, digital PCR, highly sensitive detection, quantification

## Abstract

The mutation of T790M in EGFR is a major mechanism of resistance to treatment with EGFR-TKIs. Only qualitative detection (presence or absence) of T790M has been described to date, however. Digital PCR (dPCR) analysis has recently been applied to the quantitative detection of target molecules in cancer with high sensitivity. In the present study, 25 tumor samples (13 obtained before and 12 after EGFR-TKI treatment) from 18 NSCLC patients with activating *EGFR* mutations were evaluated for T790M with dPCR. The ratio of the number of T790M alleles to that of activating mutation alleles (T/A) was determined. dPCR detected T790M in all 25 samples. Although T790M was present in all pre-TKI samples from 13 patients, 10 of these patients had a low T/A ratio and manifested substantial tumor shrinkage during treatment with EGFR-TKIs. In six of seven patients for whom both pre- and post-TKI samples were available, the T/A ratio increased markedly during EGFR-TKI treatment. Highly sensitive dPCR thus detected T790M in all NSCLC patients harboring activating *EGFR* mutations whether or not they had received EGFR-TKI treatment. Not only highly sensitive but also quantitative detection of T790M is important for evaluation of the contribution of T790M to EGFR-TKI resistance.

## INTRODUCTION

Treatment with epidermal growth factor receptor (EGFR) tyrosine kinase inhibitors (TKIs) has become the standard care for patients with non–small cell lung cancer (NSCLC) positive for activating mutations of *EGFR* [1-7]. Several methods based on the polymerase chain reaction (PCR), such as the Scorpion amplification refractory mutation system (ARMS), have been developed for *EGFR* mutation screening and have a sensitivity that allows the detection of a mutant allele fraction of at least 1% (one mutant molecule in a background of 99 wild-type molecules) [8-11]. These laboratory tests are now commercially available and are widely used to detect target *EGFR* mutations in patients for appropriate implementation of treatment with EGFR-TKIs.

Although treatment of patients positive for activating mutations of *EGFR* with EGFR-TKIs has a pronounced clinical benefit initially, such individuals inevitably develop drug resistance, within ~1 year on average [12, 13]. The mutation of threonine-790 to methionine (T790M) in EGFR is a major cause of resistance to EGFR-TKIs, accounting for ~60% of patients with resistance to these drugs [12–15]. T790M has also been detected in specimens obtained from NSCLC patients before treatment with EGFR-TKIs, with the detection rate for the mutation being dependent on the sensitivity of the technique [16-19]. These previous studies evaluated T790M only with qualitative methods, however. Digital PCR (dPCR) is based on the performance of PCR with a single template molecule and is able to detect targets in a quantitative and highly sensitive manner. This technique has recently been applied to detect target molecules in various cancer types [20-23].

We have now applied dPCR to the quantitative and highly sensitive detection of T790M in specimens obtained from NSCLC patients with activating *EGFR* mutations either before or after (or both before and after) treatment with EGFR-TKIs.

## RESULTS

### Patients and sample collection

We selected 18 patients whose cytological specimens (including adequate cancer cells) or frozen cell pellets were available from among patients diagnosed with NSCLC positive for major activating mutations of *EGFR* [L858R or deletions in exon 19 (Ex19 del)] at Kyushu University Hospital between October 2002 and December 2012. The study was approved by the Ethics Committee of Kyushu University.

The characteristics of the 18 patients analyzed in the study are shown in Table 1. All patients had adenocarcinoma positive for activating *EGFR* mutations confirmed by a conventional PCR-based method. Eleven of the 18 patients were positive for Ex19 del, and seven were positive for L858R. The T790M mutation was also detected by conventional PCR in specimens obtained from two patients (nos.12 and 13) before EGFR-TKI treatment (pre-TKI samples).

**Table 1:** Characteristics of the study patients and their history of chemotherapy

Patient no.	Sex	Age (years)*	Histological or cytological features	*EGFR* mutation	History of EGFR-TKI treatment	History of cytotoxic chemotherapy†	Best clinical response to EGFR-TKIs‡	Total period of EGFR-TKI treatment (days)
1	M	70	Adeno	L858R	GEF	Yes	PR	505
2	F	68	Adeno	Ex19 del	GEF	Yes	PR	2644
3	F	73	Adeno	Ex19 del	GEF	Yes	PR	686
4	F	70	Adeno	Ex19 del	GEF, ERL	No	SD	751
5	F	71	Adeno	L858R	GEF	No	PR	582
6	M	52	Adeno	Ex19 del	GEF, ERL	Yes	PR	527
7	F	68	Adeno	Ex19 del	ERL	Yes	PR	304
8	F	54	Adeno	Ex19 del	GEF, ERL	Yes	PR	373
9	F	68	Adeno	Ex19 del	GEF	No	PR	202
10	M	76	Adeno	Ex19 del	GEF	Yes	PR	407§
11	F	61	Adeno	L858R	GEF, ERL	No	PR	283§
12	M	79	Adeno	L858R/T790M	GEF	Yes	PD	95
13	F	36	Adeno	L858R/T790M	No	No	NA	NA
14	F	73	Adeno	Ex19 del	GEF	Yes	PR	1635
15	M	55	Adeno	Ex19 del	GEF, ERL	Yes	SD	428
16	F	51	Adeno	L858R	GEF, ERL	Yes	PR	910
17	F	41	Adeno	Ex19 del	GEF, ERL	Yes	PR	494
18	M	50	Adeno	L858R	GEF	No	PD	20

Abbreviations: Adeno, adenocarcinoma; GEF, gefitinib; ERL, erlotinib; PR, partial response; SD, stable disease; PD, progressive disease; NA, not applicable.

* Age is that at which treatment with the first EGFR-TKI was started.

† Chemotherapy from collection of the pre-TKI sample to that of the post-TKI sample.

‡ According to the Response Evaluation Criteria in Solid Tumors (RECIST).

§ These patients (nos.10 and 11) are currently receiving EGFR-TKI treatment without PD.

Seventeen patients received EGFR-TKI (gefitinib or erlotinib, or both) treatment, whereas one patient positive for T790M received only best supportive care (no anticancer treatment). Of the 17 patients treated with EGFR-TKIs, 13 (76.5%) achieved a partial response. The treatment period for EGFR-TKIs ranged from 20 to 2644 days (median, 527 days).

A total of 25 tumor samples, including 13 pre-TKI and 12 post-TKI specimens, was obtained from the 18 patients in the study. Eighteen of the samples were derived from pleural effusion, three from the primary tumor, three from peritoneal effusion, and one from pericardial effusion (Table 2). The samples consisted of 18 cytological specimens processed for Papanicolaou staining (C samples) and seven frozen cell pellets obtained from malignant effusion (F samples).

**Table 2:** Quantitative and qualitative evaluation of T790M in pre-TKI and post-TKI samples by dPCR

Pre-TKI samples*	Sampling site	T790M detection		Input DNA (ng) applied to each panel			Estimated number of target alleles applied to panels (1) + (2)			Ratio of estimated nos. of alleles (%)		T/A (%)†
		ARMS	dPCR	Control	Activating mutation	T790M	Control	Activating mutation	T790M	Activating mutation/control	T790M/control	
1–1-C	Pleural effusion	−	+	2.5	2	100	762	400	103	65.62	0.34	0.52
2–1-C	Pleural effusion	−	+	44.6	44.6	44.6	329	10	2	3.04	0.61	20.00
3–1-C	Pleural effusion	−	+	20	40	100	465	454	20	48.82	0.86	1.76
4–1-C	Pericardial effusion	−	+	14	28	100	2911	4440	87	76.26	0.42	0.55
5–1-C	Primary lesion	−	+	14	30	87.82	1901	171	56	4.2	0.47	11.18
6–1-C	Primary lesion	−	+	50	50	100	511	101	2	19.77	0.2	0.99
7–1-C	Primary lesion	−	+	17.46	17.46	17.46	1484	448	2	30.19	0.13	0.45
8–1-F	Pleural effusion	−	+	3	25	100	1124	1213	7	12.95	0.02	0.14
9–1-F	Pleural effusion	−	+	7	25	100	2871	2166	10	21.12	0.02	0.12
10–1-F	Pleural effusion	−	+	5	25	100	1852	3184	7	34.38	0.02	0.05
11–1-F	Pleural effusion	−	+	4	30	100	1498	232	13	2.06	0.03	1.69
12–1-C	Pleural effusion	+	+	5	5	5	658	42	62	6.38	9.42	147.69
13–1-C	Pleural effusion	+	+	30	60	60	2744	55	171	1	3.12	311.59
	Detection rate	15.4%	100%									
Post-TKI samples*	Sampling site	T790M detection		Input DNA (ng) applied to each panel			Estimated number of target alleles applied to panels (1) + (2)			Ratio of estimated nos. of alleles (%)		T/A (%)†
		ARMS	dPCR	Control	Activating mutation	T790M	Control	Activating mutation	T790M	Activating mutation/control	T790M/control	
1–2-C	Pleural effusion	−	+	2.5	2.5	100	1342	52	1014	3.87	1.89	48.84
2–2-F	Pleural effusion	−	+	3	30	100	931	798	899	8.57	2.9	33.84
3–2-C	Pleural effusion	+	+	40	40	40	826	1259	477	152.42	57.75	37.89
4–2-C	Pleural effusion	+	+	12	22	61.04	2283	2601	4163	62.14	35.85	57.69
5–2-C	Pleural effusion	−	+	14	30	100	1448	562	52	18.11	0.5	2.76
6–2-C	Peritoneal effusion	+	+	20	28.82	28.82	3464	3505	1175	70.22	23.54	33.52
7–2-C	Pleural effusion	−	+	14	28	58.44	1324	101	97	3.81	1.76	46.19
14–2-F	Pleural effusion	−	+	3	30	100	1485	1207	1542	8.13	3.12	38.38
15–2-C	Peritoneal effusion	−	+	8	19.36	19.36	2261	405	7	7.4	0.13	1.76
16–2-F	Pleural effusion	+	+	3	6	6	1563	1334	947	42.67	30.29	70.99
17–2-C	Peritoneal effusion	−	+	18	25	94.48	1552	2936	39	136.21	0.48	0.35
18–2-C	Pleural effusion	−	+	7.44	7.44	7.44	1574	111	2	7.05	0.13	1.84
	Detection rate	33.3%	100%									

* C denotes cytological specimen; F denotes frozen cell pellet obtained from malignant effusion. Sample designation corresponds to patient no.–1 (pre-TKI sample) or 2 (post-TKI sample)–sample type (C or F).Both pre-TKI and post-TKI samples were available for patients 1 to 7

† T/A, the ratio of the number of T790M alleles to that of activating mutation alleles.

### Evaluation of the sensitivity and specificity of nanofluidic dPCR

We examined the sensitivity and specificity of nanofluidic dPCR for detection of the T790M mutation of *EGFR* with known DNA samples corresponding to wild-type (WT) or mutant (T790M) alleles of *EGFR*. No positive signal for T790M was detected from any of the 765 chambers when up to 1.0 × 10^4^ copies of the WT allele were applied to the digital panel as a negative control (Supplementary Table 1). Application of 1.0 × 10^5^ WT copies resulted in the appearance of 0 to 4 nonspecific positive signals in eight replicates. With the use of Poisson regression analysis, we defined the cutoff number of positive signals as 3 (95% confidence interval, 0.85 to 2.64) for detection of T790M alleles in a sample including 1.0 × 10^4^ to 1.0 × 10^5^ WT alleles.

**Figure 1:**
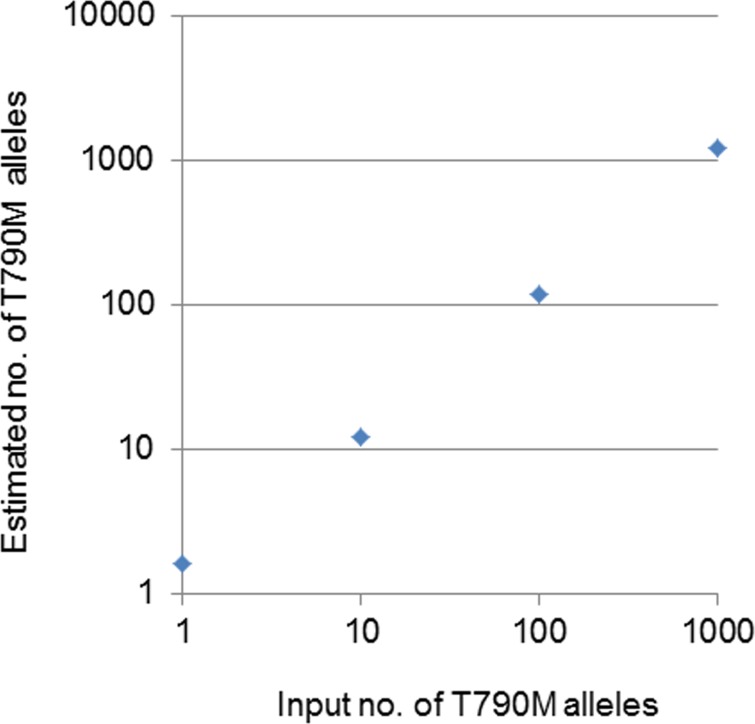
Relation between the number of input T790M alleles and the estimated number of these alleles by dPCR The input T790M alleles were added to a digital panel together with 1 × 10^4^ WT alleles.

The relation between the known number of input T790M alleles and the estimated number by dPCR was linear for 1, 10, 100, and 1000 T790M alleles in a sample including 1.0 × 10^4^ WT alleles (Figure 1). These results thus showed that the quantitative evaluation of T790M by dPCR is reliable and has a sensitivity that allows for the detection of a mutant allele fraction of 0.01% or more.

### Highly sensitive detection and quantitative evaluation of T790M by dPCR

With the use of the highly sensitive dPCR system, we then evaluated the T790M status of the 25 samples obtained from the study patients. dPCR detected T790M in all of these 25 samples, including both pre-TKI (*n* = 13) and post-TKI (*n* = 12) specimens (Table 2). In contrast, ARMS detected T790M in only 15.4% (2 of 13) and 33.3% (4 of 12) of the pre-TKI and post-TKI samples, respectively (Table 2). These data thus suggested that dPCR was able to detect T790M present in a small population of tumor cells at a frequency below the limit of detection for ARMS.

**Figure 2:**
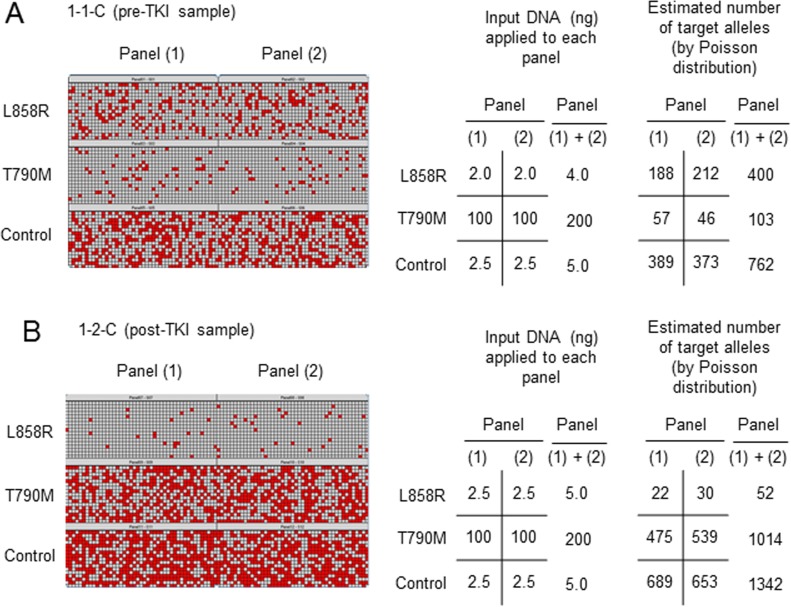
Representative results for quantitative evaluation of T790M by dPCR Two samples were obtained from the same patient (no. 1) either before (sample 1-1-C, pre-TKI) (A) or after (sample 1-2-C, post-TKI) (B) EGFR-TKI treatment. The assay was performed in duplicate [panels (1) and (2)]. Chambers with a positive reaction are indicated as red squares in a heat map for each of the 765 chambers, and the number of signals was counted by system software (raw data).

We next evaluated the frequency of T790M in pre-TKI samples. Representative results for one of these samples (1-1-C) from a patient (no. 1) with the L858R mutation of *EGFR* are shown in Figure 2A. We performed duplicate assays [panels (1) and (2)] for detection of each target allele [L858R, T790M, and control (a region of *EGFR* exon 2)]. Chambers with a positive reaction are indicated as red squares in a heat map for each of the 765 chambers, and the number of signals was counted by the system software (raw data). The number of target alleles included in the sample applied to the panels was estimated from the raw data with the use of the Poisson distribution (estimated number of target alleles), and the values for the duplicate assays were summed [(1) + (2)]. By adjusting the amount of input DNA in each reaction, we calculated the ratio of the number of activating mutation (L858R) or T790M alleles to the number of control alleles as follows: L858R/control = (400 × 5.00)/(762 × 4.00) = 65.62% (*a*), and T790M/control = (103 × 5.00)/(762 × 200) = 0.34% (*b*).

To correct for the different proportion of cancer cells and genetic heterogeneity in each sample, we calculated the ratio of the number of T790M alleles to the number of activating mutation alleles (T/A) as follows: T/A = *b*/*a* = 0.34/65.62 = 0.52%. The T/A (%) values for all 13 pre-TKI samples are presented in Table 2 and Supplementary Table 2. The results show that, with the exception of two samples (12-1-C, 13-1-C), the T790M mutation was much less frequent than activating mutations in pre-TKI specimens.

### Quantitative change in T790M frequency during EGFR-TKI treatment

To evaluate the quantitative change in T790M frequency during EGFR-TKI treatment, we also calculated T/A (%) for post-TKI samples. Representative results for a post-TKI sample (1-2-C) obtained from the same patient (no. 1) as the representative pre-TKI sample are shown in Figure 2B. We found that: L858R/control = (52 ×5.0)/(1342 × 5.0) = 3.87% (*c*), T790M/control = (1014 × 5.0)/(1342 × 200) = 1.89% (*d*), and T/A = *d*/*c* = 1.89/3.87 = 48.84%, indicating that the frequency of T790M alleles increased during EGFR-TKI treatment in this patient. The T/A (%) values for all 12 post-TKI samples are shown in Table 2 and Supplementary Table 2.

**Figure 3:**
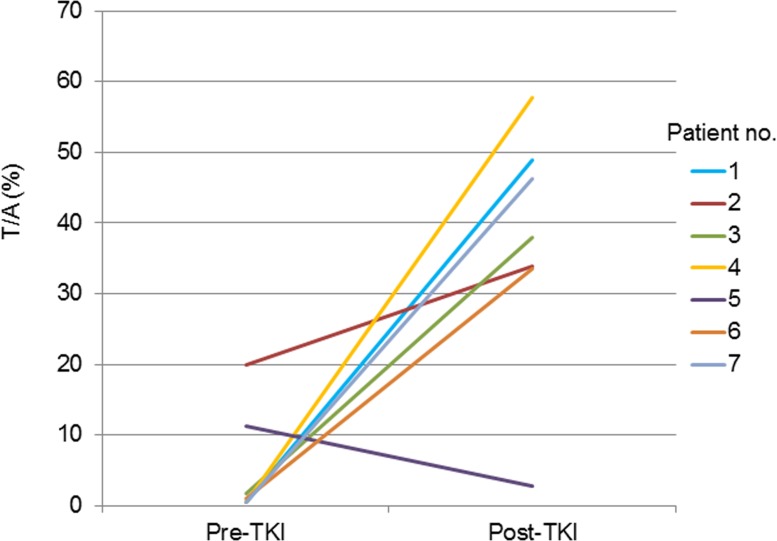
Quantitative change in the T/A ratio for the seven patients for whom both pre-TKI and post-TKI samples were available

Both pre-TKI and post-TKI samples were available for seven patients (nos.1 to 7). These seven patients received EGFR-TKI treatment for more than 10 months with substantial clinical benefit (Table 1). In six (85.7%) of these seven patients, the T/A value was greater after EGFR-TKI treatment than before (Figure 3).

## DISCUSSION

Up to 60% of individuals with NSCLC who develop resistance to EGFR-TKIs have been found to harbor the T790M secondary mutation of EGFR. This mutation has also been detected in NSCLC patients before EGFR-TKI treatment, however, with a detection rate ranging from ~30% to ~80% depending on the sensitivity of the detection technique [16-19]. In the present study, we have demonstrated the presence of the T790M mutation in all tumor specimens obtained from NSCLC patients with activating *EGFR* mutations regardless of whether the samples were obtained before or after EGFR-TKI treatment. The estimated number of control alleles applied to reaction panels for detection of T790M was limited to <1.0 × 10^5^ in all samples so as not to generate a false positive result. Given that the estimated number of T790M alleles was >3 when the applied number of control alleles was >1.0 × 10^4^ (Supplementary Table 3), the present study was performed within the reliable range for specificity of dPCR. The finding that T790M was present in all samples is therefore not a false positive.

There are several possible explanations for the higher detection rate of T790M in the present study compared with previous studies. First, the DNA analyzed in our study was evaluated by three techniques (NanoDrop, Qubit, and ARMS) for determination of an adequate and appropriate amount for application to dPCR. Given that NanoDrop overestimates the concentration of DNA, especially for tumor samples in which DNA is partially degraded, the combination of this system with measurement of double-stranded DNA (dsDNA) by Qubit is helpful for evaluation of a suitable amount of DNA for PCR [24]. The isolated DNA was also evaluated in terms of the cycle threshold (*C*_t_) value obtained by ARMS, which provides a measure of DNA quantity and suitability for PCR amplification. Second, we used cytological specimens or frozen cell pellets, which are free from the effect of formalin fixation on DNA integrity. Formaldehyde has been found to induce both the formation of cross-links between DNA and proteins as well as fragmentation of DNA [25]. In our study, the concentration of dsDNA obtained from formaldehyde-free samples was thus adequate for PCR as reflected by the low *C*_t_ values obtained by ARMS targeting the control allele (Supplementary Table 4).

In our study, T790M was detected in all 13 pre-TKI samples, and 12 of the corresponding patients were subsequently treated with EGFR-TKIs (Table 1). Ten of these 12 patients (nos. 1 to 3 and 5 to 11) had a low T/A value for the pre-TKI sample and experienced substantial tumor shrinkage during treatment with gefitinib or erlotinib (Tables 1 and 2), consistent with the previous finding that gefitinib or erlotinib was effective for the treatment of tumors estimated to have a low frequency of T790M by ARMS [26]. Administration of first-generation EGFR-TKIs such as gefitinib and erlotinib should thus not be avoided in T790M-positive patients if the T/A ratio in the pre-TKI sample is low. This conclusion is supported by a previous *in vitro* study with PC9 cells, which harbor an activating *EGFR* mutation (Ex19 del). The presence of a small proportion of cells that also harbor T790M was thus found not to substantially affect overall sensitivity to erlotinib [27]. Sensitivity to erlotinib declined, however, as the proportion of T790M-positive cells increased. In our study, the T/A ratio increased during EGFR-TKI treatment in six of the seven patients for whom both pre-TKI and post-TKI samples were available. Our results thus support the notion that tumor cells harboring T790M are present in small numbers even before EGFR-TKI treatment, and that these T790M-positive cells undergo selection and enrichment during such treatment [26]. The presence of T790M was likely responsible for the development of acquired resistance to EGFR-TKIs in these latter six patients of the present study. In the case of patient no. 5, whose T/A ratio did not increase after failure of gefitinib treatment, another resistance mechanism, such as *MET* amplification, may have been operative and responsible for resistance to the drug.

There are several limitations to our study. First, the study is retrospective and has a small sample size. Second, in most cases, cytotoxic chemotherapy was performed between the collection of pre-TKI and post-TKI samples (Table 1). The possibility therefore exists that the change in the T/A ratio was not solely attributable to selection of T790M-positive cells by EGFR-TKI treatment in such cases. Third, most specimens were obtained from metastatic lesions rather than from primary tumors, giving rise to the possibility that our results may not reflect the status of T790M in primary lesions. Finally, pre- and post-TKI samples were obtained from different sites in several cases, raising the possibility that the quantitative change in T790M frequency may have been due to intratumoral heterogeneity.

In conclusion, when highly sensitive methods such as dPCR or next-generation sequencing (NGS) are introduced into clinical practice in the near future, T790M will be detected with a high frequency in NSCLC cases positive for activating *EGFR* mutations regardless of whether the patient has been treated with EGFR-TKIs or not. Quantitative evaluation of T790M on the basis of the T/A ratio will therefore be important to determine whether T790M is likely to be responsible for EGFR-TKI resistance in such patients. Given that high-coverage NGS is also able to determine allele frequencies, a study that applies both dPCR and NGS in more patients is warranted.

## MATERIALS AND METHODS

### Sample preparation

Cells in C samples were scraped from the glass slide with a surgical blade after removal of the cover slip by overnight incubation in xylene. DNA was extracted from the cells with the use of a QIAamp FFPE Tissue Kit (Qiagen KK, Tokyo, Japan). Malignant effusion specimens had been centrifuged at 630 × *g* for 10 min at room temperature, and the cell pellets had been stored at −80°C (F samples). DNA was extracted from these samples with a QIAamp DNA Mini Kit (Qiagen KK) according to the blood and body fluid spin protocol in the manufacturer's instructions. The concentration and purity of extracted DNA in all samples were determined by spectrophotometry (NanoDrop ND-1000; Thermo Fisher Scientific, Waltham, MA). dsDNA was quantified with the use of a Quant-iT dsDNA HS Assay (Life Technologies, Carlsbad, CA) and a Qubit fluorometer (Life Technologies). The extracted DNA was stored at 4°C until analysis.

### Scorpion ARMS

We performed allele-specific PCR by Scorpion ARMS with the use of a Therascreen EGFR RGQ PCR Kit (Qiagen KK) as a conventional method to detect three targets (control, activating mutations, and T790M). We included 20 ng of DNA (as determined with NanoDrop) in each reaction. A region of exon 2 of *EGFR* was amplified as the control. ARMS reveals the number of PCR cycles necessary to detect the target molecule present at the beginning of the reaction. The cycle number at which the signal is detected above background fluorescence is defined as the cycle threshold (*C*_t_). A sample is considered to be positive for the target mutation if the difference between its mutation *C*_t_ value and its control *C*_t_ value is less than the cutoff value described in the manufacturer's instructions.

### Nanofluidic dPCR

We used a nanofluidic dPCR system (BioMark HD System; Fluidigm, South San Francisco, CA) with the Fluidigm digital chip to quantitate target DNA molecules. The digital chip delivers up to 12 mixtures of samples and PCR reagents into 12 individual panels. Each panel contains 765 independent 6-nl chambers. We estimated an appropriate amount of DNA to be applied to each panel that would yield positive signals in some but not all (*n* = 765) chambers on the basis of the *C*_t_ values of each target allele obtained with ARMS. The individual target DNA molecules become randomly distributed in the chambers after their addition. ARMS was performed in each reaction chamber targeting either control (region of exon 2), activating mutation (L858R or Ex19 del), or T790M alleles of *EGFR*. After 35 cycles of PCR, the number of signals obtained from successfully reacted chambers was counted with the Fluidigm digital PCR analysis software (raw data). The number of target alleles included in each sample was then estimated on the basis of the raw data with the use of the Poisson distribution, given that a positive reaction in a chamber may correspond to multiple target molecules.

### Statistical analysis

Statistical analysis was performed with the use of JMP version 9 software (SAS Institute, Cary, NC). We used Poisson regression analysis to decide the cutoff point for the specificity of dPCR.

## SUPPLEMENTARY FIGURES AND TABLES


