# Supplementary material for: Highly sensitive and quantitative evaluation of the EGFR T790M mutation by nanofluidic digital PCR
Source: Oncotarget. 2015 May 10;6(24):20466–73. doi: 10.18632/oncotarget.4058 (PMC4653018; doi:10.18632/oncotarget.4058)
Supplement: Supplementary file 1 [file oncotarget-06-20466-s001.pdf]

Highly sensitive and quantitative evaluation of the EGFR T790M mutation by nanofluidic digital PCR

Supplementary Material

Supplementary Table 1. Specificity of dPCR for detection of the T790M mutation of EGFR

No. of WT copies	Number of positive signals in T790M reaction	Poisson regression analysis (95% CI)
1×10^5	0	0.85 to 2.64
	0	
	0	
	3	
	0	
	2	
	4	
	3	
1×10^4	0	
	0	
	0	
1×10^3	0	
	0	
1×10^2	0	
	0	
1×10^1	0	
	0	

CI, confidence interval.

Supplementary Table 2. Detailed data for quantitative evaluation of T790M in pre-TKI and post-TKI samples by dPCR

Pre-TKI samples	Estimated no. of target alleles								
	Duplicate panels for control			Duplicate panels for activating mutation			Duplicate panels for T790M		
	Panel (1)	Panel (2)	Panels (1) + (2)	Panel (1)	Panel (2)	Panels (1) + (2)	Panel (1)	Panel (2)	Panels (1) + (2)
1-1-C	389	373	762	188	212	400	57	46	103
2-1-C	171	158	329	5	5	10	1	1	2
3-1-C	233	232	465	232	222	454	12	8	20
4-1-C	1221	1690	2911	2338	2102	4440	36	51	87
5-1-C	985	916	1901	78	93	171	23	33	56
6-1-C	247	264	511	43	58	101	2	0	2
7-1-C	701	783	1484	201	247	448	1	1	2
8-1-F	611	513	1124	676	537	1213	5	2	7
9-1-F	1491	1380	2871	1202	964	2166	7	3	10
10-1-F	874	978	1852	1588	1596	3184	2	5	7
11-1-F	729	769	1498	126	106	232	6	7	13
12-1-C	327	331	658	25	17	42	29	33	62
13-1-C	1423	1321	2744	21	34	55	64	107	171

Post-TKI samples	Estimated no. of target alleles								
	Duplicate panels for control			Duplicate panels for activating mutation			Duplicate panels for T790M		
	Panel (1)	Panel (2)	Panels (1) + (2)	Panel (1)	Panel (2)	Panels (1) + (2)	Panel (1)	Panel (2)	Panels (1) + (2)
1-2-C	689	653	1342	22	30	52	475	539	1014
2-2-F	437	494	931	406	392	798	618	281	899
3-2-C	418	408	826	639	620	1259	244	233	477
4-2-C	1062	1221	2283	1257	1344	2601	1886	2277	4163
5-2-C	711	737	1448	284	278	562	30	22	52
6-2-C	1612	1852	3464	1718	1787	3505	600	575	1175
7-2-C	674	650	1324	42	59	101	47	50	97
14-2-F	566	919	1485	660	547	1207	967	575	1542
15-2-C	1074	1187	2261	205	200	405	3	4	7
16-2-F	805	758	1563	641	693	1334	494	453	947
17-2-C	831	721	1552	1356	1580	2936	21	18	39
18-2-C	794	780	1574	53	58	111	2	0	2

Supplementary Table 3. Comparison of estimated numbers of T790M and control alleles applied to panels for detection of T790M

Pre-TKI samples	Input DNA (ng) applied to each panel		Estimated no. of control alleles applied to panels (1) + (2) for detection of control alleles (*z*)	Estimated no. of T790M alleles applied to panels (1) + (2) for detection of T790M alleles	Estimated no. of control alleles applied to panels (1) + (2) for detection of T790M alleles*
	Panel for detection of control (*x*)	Panel for detection of T790M (*y*)			
1-1-C	2.50	100.00	762	103	30480
2-1-C	44.60	44.60	329	2	329
3-1-C	20.00	100.00	465	20	2325
4-1-C	14.00	100.00	2911	87	20793
5-1-C	14.00	87.82	1901	56	11925
6-1-C	50.00	100.00	511	2	1022
7-1-C	17.46	17.46	1484	2	1484
8-1-F	3.00	100.00	1124	7	37467
9-1-F	7.00	100.00	2871	10	41014
10-1-F	5.00	100.00	1852	7	37040
11-1-F	4.00	100.00	1498	13	37450
12-1-C	5.00	5.00	658	62	658
13-1-C	30.00	60.00	2744	171	5488
Post-TKI samples	Input DNA (ng) applied to each panel		Estimated no. of control alleles applied to panels (1) + (2) for detection of control alleles (*z*)	Estimated no. of T790M alleles applied to panels (1) + (2) for detection of T790M alleles	Estimated no. of control alleles applied to panels (1) + (2) for detection of T790M alleles*
	Panel for detection of control (*x*)	Panel for detection of T790M (*y*)			
1-2-C	2.50	100.00	1342	1014	53680
2-2-F	3.00	100.00	931	899	31033
3-2-C	40.00	40.00	826	477	826
4-2-C	12.00	61.04	2283	4163	11613
5-2-C	14.00	100.00	1448	52	10343
6-2-C	20.00	28.82	3464	1175	4992
7-2-C	14.00	58.44	1324	97	5527
14-2-F	3.00	100.00	1485	1542	49500
15-2-C	8.00	19.36	2261	7	5472
16-2-F	3.00	6.00	1563	947	3126
17-2-C	18.00	94.48	1552	39	8146
18-2-C	7.44	7.44	1574	2	1574

*Estimated number of control alleles applied to panels (1) + (2) for detection of T790M was calculated as: [estimated number of control alleles applied to panels (1) + (2) for detection of control alleles (*z*)] × [input DNA applied to the panel for detection of T790M (*y*)]/[input DNA applied to the panel for detection of control (*x*)].

Supplementary Table 4. Evaluation of DNA with NanoDrop, Qubit, and ARMS

Pre-TKI samples	DNA concentration		ARMS C_t value		
	NanoDrop (ng/µL)	Qubit (dsDNA) (ng/µL)	Control	Activating mutation	T790M
1-1-C	259.10	54.60	25.8	27.15	34.87
2-1-C	22.30	0.13	31.78	34.6	ND
3-1-C	117.89	13.90	27.19	29.69	36.09
4-1-C	93.96	16.90	26.63	30.58	35.71
5-1-C	43.91	8.66	27.42	32.11	36.25
6-1-C	60.70	0.79	27.65	30.85	36.55
7-1-C	8.73	0.95	28.03	30.43	38.31
8-1-F	98.82	89.40	25.04	29.26	ND
9-1-F	253.51	95.40	24.73	27.69	39.13
10-1-F	157.34	89.40	24.8	26.71	ND
11-1-F	135.25	94.80	24.8	31.64	38.3
12-1-C	101.05	12.20	27.32	32.48	32.02
13-1-C	37.81	3.28	28.25	35.97	32.19
**Post-TKI samples**	**DNA concentration**		**ARMS C_t value**		
	NanoDrop (ng/µL)	Qubit (dsDNA) (ng/µL)	Control	Activating mutation	T790M
1-2-C	173.60	50.80	24.87	30.11	31.35
2-2-F	207.60	352.00	24.88	29.85	32.06
3-2-C	26.24	3.62	28.65	30.26	31.85
4-2-C	30.52	7.16	26.47	30.82	29.07
5-2-C	57.95	11.40	27.08	32.49	33.53
6-2-C	14.41	1.93	27.02	28.37	29.88
7-2-C	29.22	5.32	27.45	32.87	33.94
14-2-F	528.60	724.00	24.5	28.82	31.34
15-2-C	9.68	3.12	26.41	30.82	ND
16-2-F	95.63	83.00	24.59	27.32	27.39
17-2-C	47.24	6.82	27.63	27.85	36.61
18-2-C	3.72	0.98	27.61	32.28	ND

ND, not detectable.
